# Medication-Related Osteonecrosis of the Jaws and CDK4/6 Inhibitors: A Recent Association

**DOI:** 10.3390/ijerph17249509

**Published:** 2020-12-18

**Authors:** Antonia Marcianò, Gian Marco Guzzo, Matteo Peditto, Antonio Picone, Giacomo Oteri

**Affiliations:** 1 Department of Clinical and Experimental Medicine, University of Messina, 98122 Messina, Italy; antmarciano@unime.it; 2 Humanitas Centro Catanese di Oncologia, Department of Medical Oncology, 95045 Misterbianco, Italy; antonio.picone@ccocatania.it; 3 Department of Biomedical and Dental Sciences and Morphofunctional Imaging, University of Messina, 98122 Messina, Italy; gianmarco.guzzo@libero.it (G.M.G.); giacomo.oteri@unime.it (G.O.)

**Keywords:** osteonecrosis of the jaw, oral epidemiology, oral prevention, oral adverse events

## Abstract

The purpose of the present study was to estimate the prevalence of cyclin-dependent kinase (CDK) 4/6 inhibitors use among cancer patients from the medication-related osteonecrosis of the jaw (MRONJ) cohort of the University of Messina. We retrospectively reviewed the records of all patients with either intravenous bisphosphonates or denosumab-related MRONJ reported in the electronic health records of the Unit of Oral Surgery, School of Dentistry, University of Messina between the first quarter of 2018 and the first quarter 2020 to identify eligible patients. We observed six cases of MRONJ associated with CDK4/6 inhibitors concomitantly with intravenous bisphosphonates and/or denosumab in breast cancer patients. The CDK4/6 inhibitors registered were palbociclib (n = 5) and abemaciclib (n = 1). Data of cancer patients diagnosed with MRONJ in the same period (n = 10) were extracted for comparison. The comparative assessment with this group of patients showed a similar distribution of MRONJ stage ranged and clinical course after treatment. The degree of risk for osteonecrosis in patients taking these new classes of drugs is uncertain but warrants awareness and close monitoring. The role of premedication dental evaluation as a prevention strategy has been acknowledged for cancer patients about to initiate intravenous bisphosphonates and/or denosumab for treatment of bone metastasis, but additional attention should be paid to whom are assuming CDK4/6 inhibitors because of their oral adverse events.

## 1. Introduction

The discovery of various driver pathways and targeted small molecule agents/antibodies have revolutionized the management of metastatic breast cancer. Currently, the major targets of clinical utility in breast cancer include the human epidermal growth factor receptor 2 (HER2) and epidermal growth factor receptor (EGFR), vascular endothelial growth factor (VEGF) receptor, mechanistic target of rapamycin (mTOR) pathway and the cyclin-dependent kinase 4/6 (CDK4/6) pathway [1]. Palbociclib and abemaciclib are approved in combination with an aromatase inhibitor or fulvestrant for HR+ MBC. Abemaciclib is also approved as monotherapy for pretreated patients [2]. Palbociclib is a cyclin-dependent kinase (CDK) 4/6 inhibitor approved by the Food and Drug Administration (FDA) for the HR-positive, HER2-negative advanced or metastatic breast cancer in 2015 in combination with an aromatase inhibitor (AI) and letrozole [2].

Abemaciclib is another CDK4/6 kinase inhibitor but more potent against CDK4. It was approved by the FDA in 2017 for the treatment of the postmenopausal woman with the HR-positive, HER2-negative advanced or metastatic breast cancer in combination with fulvestrant [2]. The toxicity profile of the CDK4/6 inhibitors has been detailed in the clinical trials for each drug in the class (palbociclib-PALOMA; ribociclib-MONALEESA; abemaciclib-MONARCH) and postmarketing reports [3,4,5,6]. Stomatitis was a common (15.3%) adverse reaction reported in palbociclib-treated patients in randomized clinical trials. Stomatitis includes the following: aphthous stomatitis, cheilitis, glossitis, glossodynia, mouth ulceration, inflammation of the mucous membrane, oral pain, oropharyngeal disorder, oropharyngeal pain and stomatitis [3]. Stomatitis/mucositis could eventually be implicated in medication-related osteonecrosis of the jaw (MRONJ) development due to breaking of the mucosal lining in the mouth and exposure of the underlying bone to bacteria [7,8]. The purpose of the present study was to estimate the prevalence of CDK4/6 inhibitors use among the cancer patients from the MRONJ cohort of the University of Messina.

## 2. Materials and Methods

The described work has been carried out in accordance with the ethical standards of the institutional and/or national research committee and with the 1964 Declaration of Helsinki and its later amendments or comparable ethical standards. For the characterization of this study group, we retrospectively reviewed the records of all patients with either intravenous bisphosphonates or denosumab-related MRONJ reported in the electronic health records (EHRs) of the Unit of Oral Surgery, School of Dentistry, University of Messina between the first quarter of 2018 and the first quarter 2020. Inclusion criteria were: (a) MRONJ diagnosis performed on the basis of the Italian Society of Oral Pathology (SIPMO) definition and staging system, (b) CDK4/6 inhibitor treatment and (c) zoledronic acid and/or denosumab use for bone metastasis treatment. In the SIPMO staging system, the clinical signs and symptoms (pain and inflammation/infection) were used to distinguish between the asymptomatic and symptomatic manifestations within the same stage [9]. In the clinical routine of the Osteonecrosis of the Jaw Treatment Center, School of Dentistry, University of Messina, this classification is currently adopted since it helps to better define the therapeutic needs of patients related to exacerbations of the infectious process and treatment is planned in accordance [10]. All patients’ medical records (including radiographic findings such as orthopanthomography and/or cone beam computed tomography for MRONJ diagnosis confirmation and staging) have been evaluated. The patients’ anamnestic features (primary disease, comorbid conditions, i.e., hypertension, diabetes and lipid disorders) and the local risk factors were analyzed. Suspected medications (zoledronic acid and/or denosumab) and their cumulative dose as well as the concomitant cancer medications supposed to have a synergic effect in MRONJ development were analyzed. Anatomic location and numbers of exposed maxillary necrotic bone areas were described and evaluated. MRONJ management was investigated and divided into two types of intervention: (1) medical treatment and (2) surgery. The clinical course of MRONJ was registered, and outcome has been stratified into the following groups: healed (with the addition of the specific case spontaneous expulsion of sequestrum), partially healed, unchanged and progressive. Data of cancer patients diagnosed with MRONJ in the same period were extracted for comparison.

## 3. Results

This report described six consecutive cases of MRONJ in patients assuming CDK4/6 inhibitors concomitantly with intravenous bisphosphonates and/or denosumab among a total of 16 patients diagnosed with MRONJ between the first quarter of 2018 and the first quarter 2020 at the Unit of Oral Surgery, School of Dentistry, University of Messina in the same period. Characteristics of the six CDK4/6 inhibitors related MRONJ and of the 16 cancer patients enrolled for the purpose of comparison are illustrated in Table 1.

All the six cases of MRONJ associated with CDK4/6 inhibitors assumed concomitantly with intravenous bisphosphonates and/or denosumab had breast cancer. Three patients received denosumab and two patients received zoledronic acid, whereas one patient was subsequently treated with zoledronic acid and denosumab. Average duration of therapy at time of MRONJ diagnosis was 24 months. On average, MRONJ occurred after 35 administrations of zoledronic acid and after 10 administrations of denosumab. The CDK4/6 inhibitors registered were palbociclib (n = 5) and abemaciclib (n = 1). No cases of MRONJ that occurred during ribociclib administration were registered in the EHRs of the Unit of Oral Surgery, School of Dentistry, University of Messina in the study period. Data of cancer patients diagnosed with MRONJ in the same period were extracted for comparison. Two patients had breast cancer, five had prostate cancer and three had multiple myeloma. Among these patients, six received denosumab and nine received zoledronic acid, whereas one patient was initially treated with zoledronic acid and then switched to denosumab therapy. On average, MRONJ occurred after 30.3 administrations of zoledronic acid and 19.4 administrations of denosumab. The cancer medications reported for the other MRONJ cases were: abiraterone (n = 2), leuprorelin (n = 1), GnRH agonist (n = 1) and radium-223 for metastatic prostate cancer, fulvestrant (n = 1) and everolimus (1) for advanced-stage breast cancer and lenalidomide (n = 3) to treat multiple myeloma. Among patients enrolled in the MRONJ cohort, the most frequent comorbidity was heart disease (n = 11), followed by lipid disorders (n = 6), osteoporosis (n = 5), diabetes (n = 3) and diabetes (n = 3). MRONJ features such as disease stage, location, treatment, surgical procedure and outcome are summarized in Table 2.

**Figure 1:**
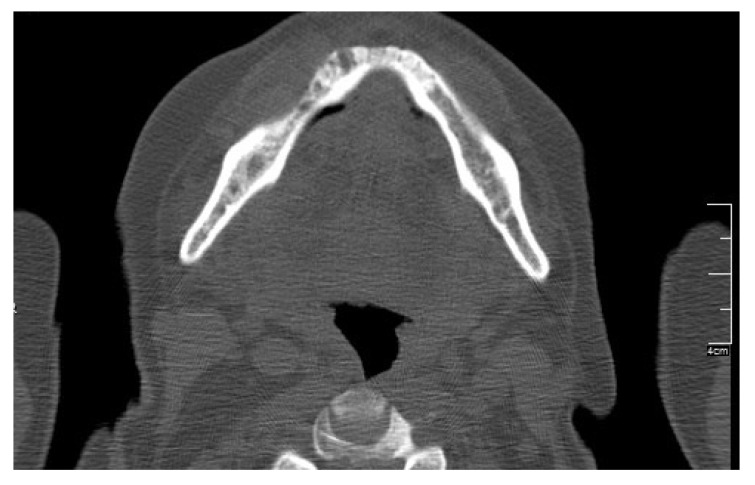
Computed tomography showing mandibular medication-related osteonecrosis of the jaw.

**Figure 2:**
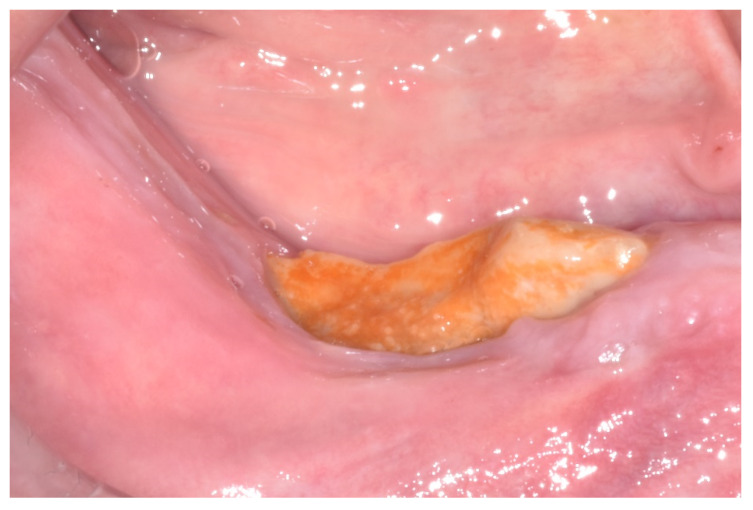
Clinical presentation of mandibular medication-related osteonecrosis of the jaw.

**Figure 3:**
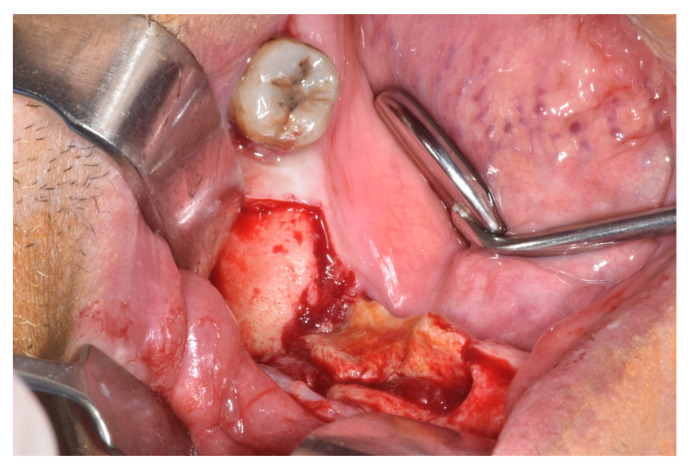
Intraoperative situation with necrotic bone sequestrum.

**Figure 4:**
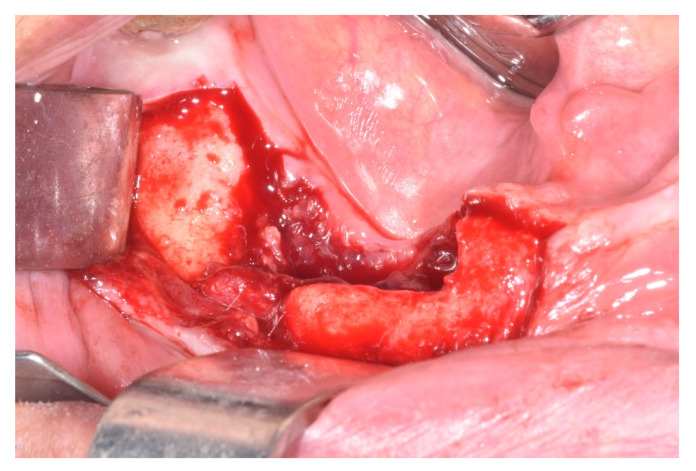
Removal of necrotic bone sequestrum revealing inflammatory reaction.

**Figure 5:**
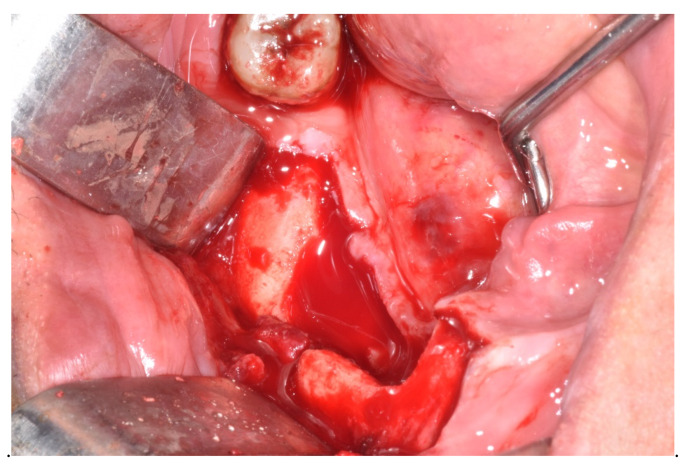
Healthy bleeding bone surface.

**Figure 6:**
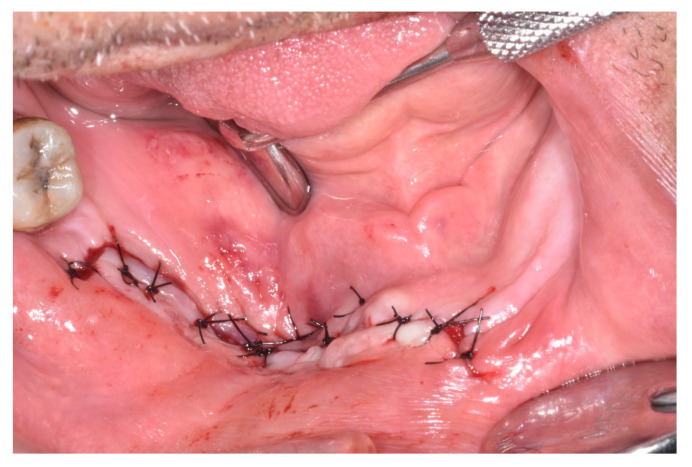
Primary healing.

**Figure 7:**
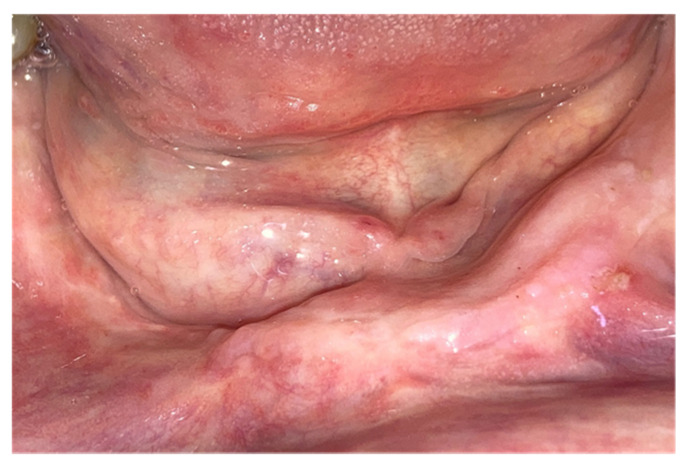
One-month follow-up.

All MRONJ stages except for stage IIIa were included: stage Ia (n = 1), Ib (n = 2), stage IIa (n = 1), IIb (n = 1) and stage III b (n = 1). The comparative assessment with this group of patients showed a similar distribution of MRONJ stages ranged between Ia (n = 1), Ib (n = 3), IIa (n = 2), IIb (n = 5) and IIIb (n = 5). The adopted therapeutic strategies have been reported for patients taking CDK4/6 inhibitors and for other cancer patients of the cohort as well. In total, 10 patients were eligible for surgical treatment (Figure 1, Figure 2, Figure 3, Figure 4, Figure 5, Figure 6 and Figure 7) whereas for 6 patients, surgery was contraindicated, and disease control was obtained through medical treatment only.

The course of the disease in patients with CDK4/6 inhibitor-associated MRONJ and cancer from the cohort was registered. Six patients healed completely. Two patients healed partially with symptoms improvement but unchanged clinical manifestation. One patient remained stable with bone exposure and prolonged local antiseptic therapy, but the disease worsened in one patient. Among the six patients with CDK4/6 inhibitor-associated MRONJ, five patients healed completely. In one of these cases, the spontaneous exfoliation of the sequestrum after medical therapy was observed. In one patient, the disease worsened, developing extraoral fistula.

## 4. Discussion

MRONJ prevalence in cancer patients stands around 2.09% in breast cancer, 3.8% in prostate cancer and 5.16% in multiple myeloma patients [11]. In the considered cohort, half of the patients with osteonecrosis had breast cancer. Although comorbid conditions under effective control were not a risk factor predisposing for the development of MRONJ, the prevalence of comorbid conditions in MRONJ patients was evaluated in view of the average age of the cohort, and the results confirmed the increasing prevalence of multimorbidity among older adults [12]. The increasing awareness of the risk for MRONJ among patients assuming novel molecules for cancer treatment [13] and/or multiple medications with synergy effect has already been described [14,15]. It has been reported that the combination of bisphosphonates and antiangiogenic factors may increase the risk of MRONJ [16]. Osteonecrosis of the jaw associated with multitargeted kinase inhibitors of the VEGF and platelet-derived growth factor (PDGF) receptors has been described in a review by Vigarios et al. [17]. Seven cases of MRONJ associated with targeted therapies (TTs) as monotherapy and in combination with antiresorptives have been described in literature [14]. Several case reports and patient series have described the occurrence of MRONJ in patients taking protein kinase inhibitors in association with bisphosphonates as well as in patients without a history of drugs related to the event osteonecrosis of the jaws [18,19,20,21,22,23]. A case of MRONJ in a patient receiving imatinib plus bisphosphonates has been reported, which recurred after denosumab administration [24]. The case of a woman aged 59 years with metastatic colorectal cancer was reported describing a necrotic bone exposure in the upper jaw with pain and soft tissue inflammation after 22 months of regorafenib treatment [25]. MRONJ was observed in a 51-year-old woman with medullary thyroid cancer receiving cabozantinib, a new tyrosine kinase inhibitor with antiangiogenic activity [26]. There was also a case of MRONJ in a cancer patient receiving lenvatinib with no history of antiresorptive treatment [27]. Data on oral toxicities induced by the recent Food and Drug Administration (FDA)- and European Medicines Agency (EMA)-approved cancer medications suggest that even if a causal relationship is difficult to establish, MRONJ could be the consequence of anticancer therapies acting as a contributing factor to the occurrence of the disease in patients exposed to intravenous bisphosphonates or denosumab. Based on available randomized trials, data on MRONJ development during treatment with palbociclib and abemaciclib are not available since this event was not reported. Nevertheless, the studies evaluating palbociclib showed a low occurrence of stomatitis, being 30% vs. 14% and 28% vs. 13% in the PALOMA-2 trial and in the phase III PALOMA-3 trial, respectively [28]. In the MONARCH-2 trial, the incidence of all-grade oral mucositis (OM) during abemaciclib administration was low (15% vs. 10%), being 1% ≥ G3 OM [29,30]. Understanding the debilitating side effects, including mucosal injury and MRONJ as a secondary effect of anticancer treatment, is an important area of clinical research. Clinical presentation and severity of reported symptoms are influenced by patient-related risk factors, systemic and local (oral), and by patients’ subjective evaluation which within an overall record of primary disease and adverse events can be underestimated. In the light of these considerations, as already reported in the case of several targeted therapies (TTs) administered in combination with antiresorptives [8], also for CDK4/6, the risk of developing MRONJ due to oral toxicity may be increased.

Between the first quarter of 2018 and the first quarter of 2020, out of 16 cancer patients diagnosed with MRONJ at the Unit of Oral Surgery, School of Dentistry, University of Messina, 6 consecutive cases have been reported in patients assuming cyclin-dependent kinase (CDK) 4/6 inhibitors. We interestingly noticed that approximately 37.5% of the reported cases of MRONJ were breast cancer patients assuming cyclin-dependent kinase (CDK) 4/6 inhibitors. This finding was surprising.

As an observational study type, our investigation has a lot of weaknesses because of the fact that within our EHRs database, only MRONJ cases are reported, so it is not possible to estimate the true incidence of MRONJ in subjects taking these medications, because of the limited number of the subjects in this study cohort, and moreover, because cancer patients with MRONJ are usually treated with multiple drugs concomitantly or subsequently.

Despite its limitations, this is the first study, to our knowledge, to estimate the prevalence of CDK4/6 inhibitor use among MRONJ patients, and we would like to call for more detailed reporting of oncological therapeutic schemes in the adverse reaction reporting forms together with the suspected drugs.

## 5. Conclusions

The degree of risk for MRONJ in patients taking these new classes of cancer medications is uncertain but warrants awareness and close monitoring. As already reported in the case of several targeted therapies (TTs) administered in combination with antiresorptives, also for CDK4/6, the risk of MRONJ may be increased.

Oral/dental follow-up with personalized schedule should be planned for patients assuming CDK4/6 inhibitors and antiresorptive medications since these cancer treatments may represent an additional risk factor for the occurrence of medication osteonecrosis of the jaws [31].

## Figures and Tables

**Table 1:** Characteristics of patients with cyclin-dependent kinase (CDK) 4/6 inhibitor-associated medication-related osteonecrosis of the jaw (MRONJ) and cancer in the cohort.

	Case 1	Case 2	Case 3	Case 4	Case 5	Case 6	Total of the CDK4/6 Inhibitors Cases n = 6	Total of the MRONJ Cases in the Cohort n = 16
Age (year)								
	63	47	61	60	57	58	58 (average) 47–63 (DS)	69 (average) 47–87 (DS)
Gender								
Female	x	x	x	x	x	x	6	9
Male								7
Cancer								
Breast	x	x	x	x	x	x	6	8
Prostate								5
Multiple Myeloma								3
Comorbidity								
Heart disease		x			x		2	11
Osteoporosis		x					1	5
Diabetes				x			1	3
Lipis disorders	x	x					2	6
Protocol								
Zometa	x					x	2	9
Denosumab		x	x		x		3	6
Zometa + denosumab				x			1	1
Duration of therapy (months)								
	68	8	19	19	3	27	24 (average)	27, 38 (average)
Cancer medication at MRONJ diagnosis								
Palbociclib	x	x		x	x	x	5	
Abemaciclib			x				1	
223 Radium								1
Lenalidomide								3
Abiraterone								2
Leuprorelin								1
Fulvestrant								1
Everolimus								1
GhRH agonist								1

**Table 2:** MRONJ features.

	Case 1	Case 2	Case 3	Case 4	Case 5	Case 6	Total of the CDK4/6 Inhibitors Cases n = 6	Total of the MRONJ Cases in the Cohort n = 16
Location								
Upper jaw		x	x				2	6
Lower jaw	x			x	x	x	4	8
Both jaws								2
Stage								
Ia					x		1	1
Ib		x				x	2	3
IIa			x				1	2
IIb				x			1	5
IIIa								
IIIb	x						1	5
Treatment								
Medical	x		x		x		3	6
Surgical		x		x		x	3	10
Procedure								
Debridement								3
Sequestrectomy						x	1	1
Saucerization								3
Submarginal resection		x		x			2	3
Outcome								
Spontaneous expulsion of sequestrum			x				1	
Healed		x		x	x	x	4	
Partially healed								
Unchanged								
Progressive	x						1	

## Abbreviations

CDK4/6	Cyclin-dependent kinase 4/6
HER2	Human epidermal growth factor receptor 2
EGFR	Epidermal growth factor receptor
VEGF	Vascular endothelial growth factor
mTOR	Receptor, mechanistic target of rapamycin
MRONJ	Medication-related osteonecrosis of the jaw
EHRs	Electronic health records
SIPMO	Italian Society of Oral Pathology
PDGF	Platelet-derived growth factor
FDA–EMA	Food and Drug Administration and European Medicines Agency
OM	Oral mucositis
TTs	Targeted therapies
GhRH	Growth Hormone Releasing Hormone
